# Population pharmacokinetic analysis and dosing optimization of polymyxin B in critically ill patients

**DOI:** 10.3389/fphar.2023.1122310

**Published:** 2023-03-29

**Authors:** Danhong Liang, Zhi Liang, Guoliang Deng, Anfen Cen, Dandan Luo, Chen Zhang, Suiqin Ni

**Affiliations:** ^1^ School of Biology and Biological Engineering, South China University of Technology, Guangzhou, Guangdong, China; ^2^ Department of Pharmacy, The Second Affiliated Hospital, School of Medicine, South China University of Technology, Guangzhou, Guangdong, China

**Keywords:** polymyxin B, critically ill patients, population pharmacokinetics, albumin levels, dosing optimization

## Abstract

**Objectives:** Since the global broadcast of multidrug-resistant gram-negative bacteria is accelerating, the use of Polymyxin B is sharply increasing, especially in critically ill patients. Unsatisfactory therapeutic effects were obtained because of the abnormal physiological function in critically ill patients. Therefore, the determination of optimal polymyxin B dosage becomes highly urgent. This study aimed to illustrate the polymyxin B pharmacokinetic characteristics by defining the influencing factors and optimizing the dosing regimens to achieve clinical effectiveness.

**Methods:** Steady-state concentrations of polymyxin B from twenty-two critically ill patients were detected by a verified liquid chromatography-tandem mass spectrometry approach. The information on age, weight, serum creatinine, albumin levels, and Acute Physiology and Chronic Health Evaluation-II (APACHE-II) score was also collected. The population PK parameters were calculated by the non-parametric adaptive grid method in Pmetrics software, and the pharmacokinetic/pharmacodynamics target attainment rate was determined by the Monte Carlo simulation method.

**Results:** The central clearance and apparent volume of distribution for polymyxin B were lower in critically ill patients (1.24 ± 0.38 L h^-1^ and 16.64 ± 12.74 L, respectively). Moreover, albumin (ALB) levels can be used to explain the variability in clearance, and age can be used to describe the variability in the apparent volume of distribution. For maintaining clinical effectiveness and lowering toxicity, 75 mg q12 h is the recommended dosing regimen for most patients suffering from severe infections.

**Conclusion:** This study has clearly defined that in critically ill patients, age and ALB levels are potentially important factors for the PK parameters of polymyxin B. Since older critically ill patients tend to have lower ALB levels, so higher dosages of polymyxin B are necessary for efficacy.

## 1 Introduction

Over the last decade, more pathogenic bacteria have developed resistance, making many first-line antibiotics ineffective against infections (Mohapatra et al., 2021). Polymyxins were used extensively as salvage therapeutic agents for combating multidrug-resistant gram-negative bacteria (MDR-GNB) infections (Falagas et al., 2021). The critically ill patients represent a unique population who exhibit higher APACHE-II scores, lower serum albumin levels and hemodynamic instability, as well as more significant differences in creatinine clearance (Roberts et al., 2014). These specific physiological features may cause changes in drug pharmacokinetic (PK), such as drug clearance and apparent volume of distribution, which lead to insufficient antibiotics concentrations in sites of infection and are associated with prolonged hospital stay and poor prognosis (Udy et al., 2013). Previous PK/PD studies have shown that the rate of reaching the standard polymyxin B exposure AUC at a typical clinical dose is low, approximately 54.3% (Ye et al., 2022) in critically ill patients. However, the exact causes have never been defined. Research has shown that body weight (WT) and creatinine clearance (CCR) are two essential factors involving PK parameters in patients (Avedissian et al., 2019), while other potential factors, such as age and ALB, on PK parameters have not been explored deeply.

In this study, 22 adult critically ill patients were given intravenous polymyxin B administration, and a population pharmacokinetic (PPK) model was created by the non-parametric adaptive grid (NPAG) algorithm. The relationship between PK parameters and covariates (WT, CCR, ALB, age, and APACHE-II) was analyzed by linear regression. A covariate model would be built for further confirmation. For finding optimal dosing regimens of polymyxin B, the Monte Carlo approach was performed to estimate the probabilities of target attainment (PTA) for clinical dosing regimens in the simulated group with modifying age and various ALB concentration ranges.

## 2 Methods

### 2.1 Patients and ethics

We gather data from critically ill patients (≥18 years old) who were given intravenous drip polymyxin B treatment with blood concentration monitoring at the hospital from November 2021 to September 2022. The attending physician decided to administer polymyxin B. At the same time, patients who received continuous renal replacement therapy (CRRT) (n = 10) or extracorporeal membrane oxygenation (ECMO) (n = 2) at the time of blood collection were excluded. Patients’ information, including gender, age, WT, serum creatinine (Scr), CCR, ALB, APACHE-II, infection diagnosis, and pathogenic bacteria, were obtained from the electronic system for medical records. CCR was generated by adopting the Cockcroft—Gault equation with WT and age (Cucci et al., 2022). The Ethical Committees of the Second Affiliated Hospital of the South China University of Technology authorized the study (K-2022-020-01).

### 2.2 Polymyxin B delivery and sampling

Patients were treated with polymyxin B, and plasma samples were collected ≥ 48 h after the treatment. Each patient contributed clinical samples, of which 37.5% (n = 24) were sampled with troughs (within 1 h before administration), 35.9% (n = 23) were sampled with peaks (within 1 h after the end of the infusion), and 26.6% (n = 17) were sampled at 6-8 h after the infusion. The dosing regimens of polymyxin B were a loading dose of 100 mg–150 mg and a maintenance dose of 50 mg–75 mg q12h, and the infusion duration was about 1–2 h. In preparation for analysis, samples were centrifuged for 10 min at 3000 r min^-1^ after collection, and the upper plasma after centrifugation was stored at −20°C.

### 2.3 Polymyxin B concentration measurements

According to previous studies, total polymyxin B1, polymyxin B2, and polymyxin B1-Ile concentrations in human blood plasma were quantified using LC-MS/MS (Meng et al., 2016; Hee et al., 2017). The standard used in our experiments was the USP standard with the batch number of R104F0. The chromatographic column was the Kinetex C18 (3 μm × 2.1 mm×100 mm. Phenomenex). A: B mobile phase was 0.1% formic acid to acetonitrile. With changes in the linear velocity ratio, gradient elution was as follows: 15% −15% B, 0–2.0 min; 15%–10% B, 2.01–4.0 min; 10%–30% B, 4.01–5.0 min; 15% B, 5.01 min; and 15%–15% B, 5.02–10 min. The flow rate was constrained to 0.8 mL min^-1^, and the column temperature was set to 40°C. The parent/daughter ion mass ratios used in the mass spectrometry were m/z 602.6/241.2 (polymyxin B1 and B1-Ile) and m/z 595.6/202.1 (polymyxin B2). Plasma samples (100 μL) were collected from centrifuged blood samples, mixed with 500 μL precipitant (acetonitrile with 6.67% formic acid) and vortexed well for 1 min. The lower limit of quantification (LLOQ) of the bioanalytical method used for quantifying polymyxin B1, B2, and B1-Ile concentrations was 0.19, 0.02 and 0.02 mg L^-1^, respectively. The linear relationships of polymyxin B1, B2, and B1-Ile concentrations in the range of 0.19–24.13 mg L^-1^, 0.02–2.7 mg L^-1^ and 0.02–2.38 mg L^-1^ were considered good (R^2^ = 0.99677, R^2^ = 0.99656, R^2^ = 0.99484) and the accuracy was within 95.3%–105.3%, 92.2%–104.8%, and 95.8%–105.4%. The RSD (%) values for the intra- and inter-day precision (n = 5) were within 2.47%–5.21%, 1.98%–6.36%, and 3.15%–6.16%, and the recovery rates were 92.89%–106.19%, 86.16%–99.94%, and 90.01%–102.30%. Finally, 200 μL of the supernatant from the centrifugation of the combination at 14,000 r min^-1^ for 10 min was employed to measure the amounts of polymyxin B.

### 2.4 PK analysis

In order to analyze the total concentrations of polymyxin B, Pmetrics version 1.9.7 (Laboratory of Applied Pharmacokinetics and Bioinformatics, Los Angeles, CA) was used by R version 4.1.3 (Neely et al., 2012). PK parameters were evaluated by a one-compartment model and a two-compartment model. The multiplicative error model (SD ×gamma), which requires the observation concentration values to be weighted by the reciprocal of the error, was used to calculate the error structure model. Gamma is the process noise that results from incorrectly specified sampling time, dosage, etc. SD is the standard deviation of the observed Cobs, calculated by the formula SD = C_0_ + C_1_ × C_obs_ + C_2_× C_obs_^2^ + C_3_ ×C_obs_^3^, where C_0_, C_1_, C_2_, and C_3_ are scientifically set to 0.1, 0.15, 0, and 0, roughly. The gamma value at the start was set to 5. The model fit indexes (negative 2 log likelihood, -2LL; the Akaike Information Criterion, AIC; the Bayesian Information Criterion, BIC) and the improvement in the goodness of fit plots (fit value, R^2^) were the model’s evaluation metrics. A standard two-compartment PK model can be described in the model building process by the following differential Eqs 1, 2.

$$\frac{dX_{1}}{dt}=RateIV+\left(\frac{Q}{V_{p}}\right)\times X_{2}-\left(\frac{CL}{V_{c}}+\frac{Q}{V_{c}}\right)\times X_{1}$$ (1)

$$\frac{dX_{2}}{dt}=\left(\frac{Q}{V_{c}}\right)\times X_{1}-\left(\frac{Q}{V_{p}}\right)\times X_{2}$$ (2)

Rate IV represents the rate of intravenous infusion. The central ventricular distribution volume (Vc) and central ventricular clearance (CL) were the fundamental PK parameters for the one-compartment model. In contrast, peripheral ventricular distribution volume (Vp) and inter-compartmental clearance (Q) were added for the two-compartment model. During covariate model construction, we evaluated covariates that might be associated with PK parameters, including age, WT, CCR, ALB, and APACHE-II score. The application of linear regression was to assess the strength of the correlations between variables and PK parameters. The covariate inclusion criteria were set as the amount of change in -2LL was more significant than 6.63 (χ ^2^ test, *p* < 0.01, df = 1) or the population fit value (R^2^ pop) was improved from the initial model. The diagnostic plot of goodness-of-fit and the visual predictive check (VPC) were used for model visual assessment. The bootstrap method and the data partitioning (dividing the entire dataset randomly into 80% and 20% parts) were utilized to gauge the model’s stability and accuracy.

### 2.5 Simulations

Combined with population PK/PD feature parameters of antibiotics medicines, the PTA of PK/PD target for a dosing regimen at a predetermined minimum inhibitory concentration (MIC) was performed by Monte Carlo simulation. According to the *2019 International Consensus Guidelines* (Tsuji et al., 2019), the target of *f*AUC/MIC for polymyxins was around 20, and the unbound fraction was determined to be 0.42 (Sandri et al., 2013). Thus AUC/MIC ≥ 50 was set as the efficacy target, as well as the MIC range was set at 0.25–4 mg L^-1^ (Satlin et al., 2020). Additionally, considering the nephrotoxicity, AUC_ss, 24h_ >100 mg∙h∙L^-1^ was chosen as an upper limit of the efficacy target (Wang et al., 2021; Yang et al., 2022). In order to take the different ALB levels into account, the data has been divided into three groups based on the clinical guidelines, including the ultra-low ALB level group (21.3–24.9 g L^-1^), the low ALB level group (25–34.9 g L^-1^), and the normal ALB level group (35 –41.5 g L^-1^). At the same time, the data has also been divided into three parts according to age, including the 5th percentile (34 years), median (68 years), and 95th percentile (93 years) values. We simulated the likelihood of achieving the AUC/MIC ≥ 50 or AUC_ss,24h_ > 100 mg∙h∙L^-1^ under various dosing regimens of polymyxin B by Pmetrics package (n = 1000). The regimens were set as follows: (Ⅰ) 100 mg q12 h; (Ⅱ) 75 mg q12 h; (Ⅲ) 60 mg q12 h; (Ⅳ) 50 mg q12h; (Ⅴ) 40 mg q12 h.

## 3 Results

### 3.1 Patient characteristics

The study included twenty-two participants, with sixty-four blood concentration measurements. Most patients (95%) had lung or bloodstream infections, and nearly all of the microorganisms that caused those illnesses were bacteria (*Acinetobacter baumannii, Pseudomonas aeruginosa, and Klebsiella pneumoniae*) resistant to carbapenem antibiotics. Other statistical information about the patients was shown in Table 1 **.**

**TABLE 1:** Characteristics of patients treated with polymyxin B.

Characteristic	Values^a^
Age(year)	68(31– 94)
Gender	Male 17(77.3)
	Female 5(22.7)
Weight(kg)	60(50–80)
APACHE-II	21.5(15–46)
Baseline Scr (umol∙L^-1^)	75(33–125)
Baseline CCR (ml∙min^-1^)^b^	68.29(34.05–192.59)
Baseline ALB (g∙L^-1^)	31.45(23.1–41.5)
Infection type	
Pulmonary Infection	11(50.0)
Pulmonary infection combined with sepsis/septicemia	10(45.5)
Intracranial infection	1(4.5)
Infection with pathogenic bacteria:	
*Pseudomonas aeruginosa*	3(13.6)
*Klebsiella pneumoniae*	2(9.1)
*Acinetobacter* baumannii	11(50.0)
Co-infection with 2 or more pathogenic bacteria	6(27.3)

a Values are median (range) or No. (%).

b Creatinine clearance formula (Cockcroft-Gault): CCR (mL min^−^1) = [(140 - age) × WT(kg)/(72 × Scr (μmol L^−1^)/88.4], all women according to the calculation results × 0.85.

### 3.2 PPK model

**FIGURE 1:**
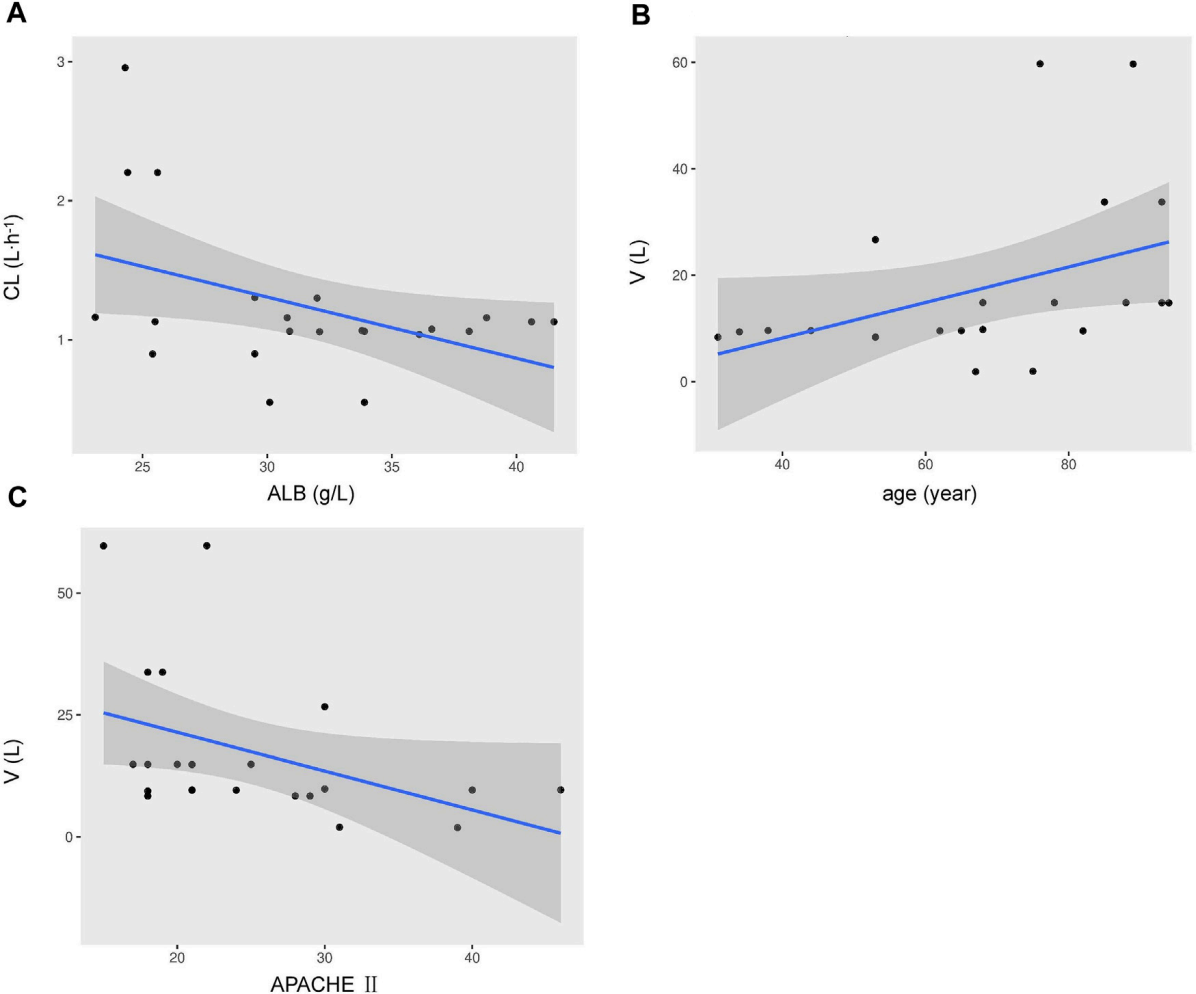
Covariates relationship using a two-compartment base model of individual polymyxin B apparent clearance estimates versus ALB **(A)**, the volume of distribution versus age **(B)**, the volume of distribution versus APACHE-II scores **(C)**. Shadow area, 95% confidence interval.

One- and two-compartment models were considered, while -2LL was used to set the base model. For polymyxin B, compared to the one-compartment model, the two-compartment model performed better (-2LL decreased by 43, decrease value > 6.63, *p* < 0.01), and the changes in other indicators were shown in Table 2. Therefore, a two-compartment model was considered for the base model. In the model 2, the relationship between covariates (age, WT, CCR, ALB, and APACHE-II) and PK model parameters was envisioned by scatter plots. A correlation was found between Vc and age (R^2^ = 0.17), as well as Vc and APACHE-II (R^2^ = 0.17), while a correlation was found between CL and ALB (R^2^ = 0.20), which was shown in Figure 1. CCR and WT were excluded because CCR had a smaller correlation coefficient with CL (R^2^ = 0.01) and WT with V (R^2^ = 0.01).

**TABLE 2:** **C**hanges in fitted values brought on by the construction of base model and stepwise covariate models^a^.

Model	Compartment	Parameters	-2LL	AIC	BIC	R^2^_pop_	R^2^_post_
1	1	Vc, CL	188.6	195.0	201.0	0.466	0.812
2	2	Vc, CL, Q, Vp	145.6	156.6	166.4	0.485	0.930
3	2	Vc, CL_0_*(ALB/31.45)^^(−0.95)^,Q, Vp	145.3	156.3	166.1	0.559	0.941
4	2	V0*(age/68)^^0.95^, CL, Q, Vp	139.9	151.0	160.7	0.486	0.926
5	2	V0*(APACHE-II/21.5)^^(−0.95)^, CL,Q, Vp	143.7	154.7	164.5	0.485	0.94
6	2	V0*(age/68)^^0.95^,CL0*(ALB/31.45)^^(−0.95)^, Q, Vp	138.9	150.0	159.7	0.573	0.925

a Abbreviations: R^2^_pop_, R^2^ values for the population’s observed versus predicted plot; R^2^_post_, R^2^ values for the individual’s observed versus predicted plot.

**FIGURE 2:**
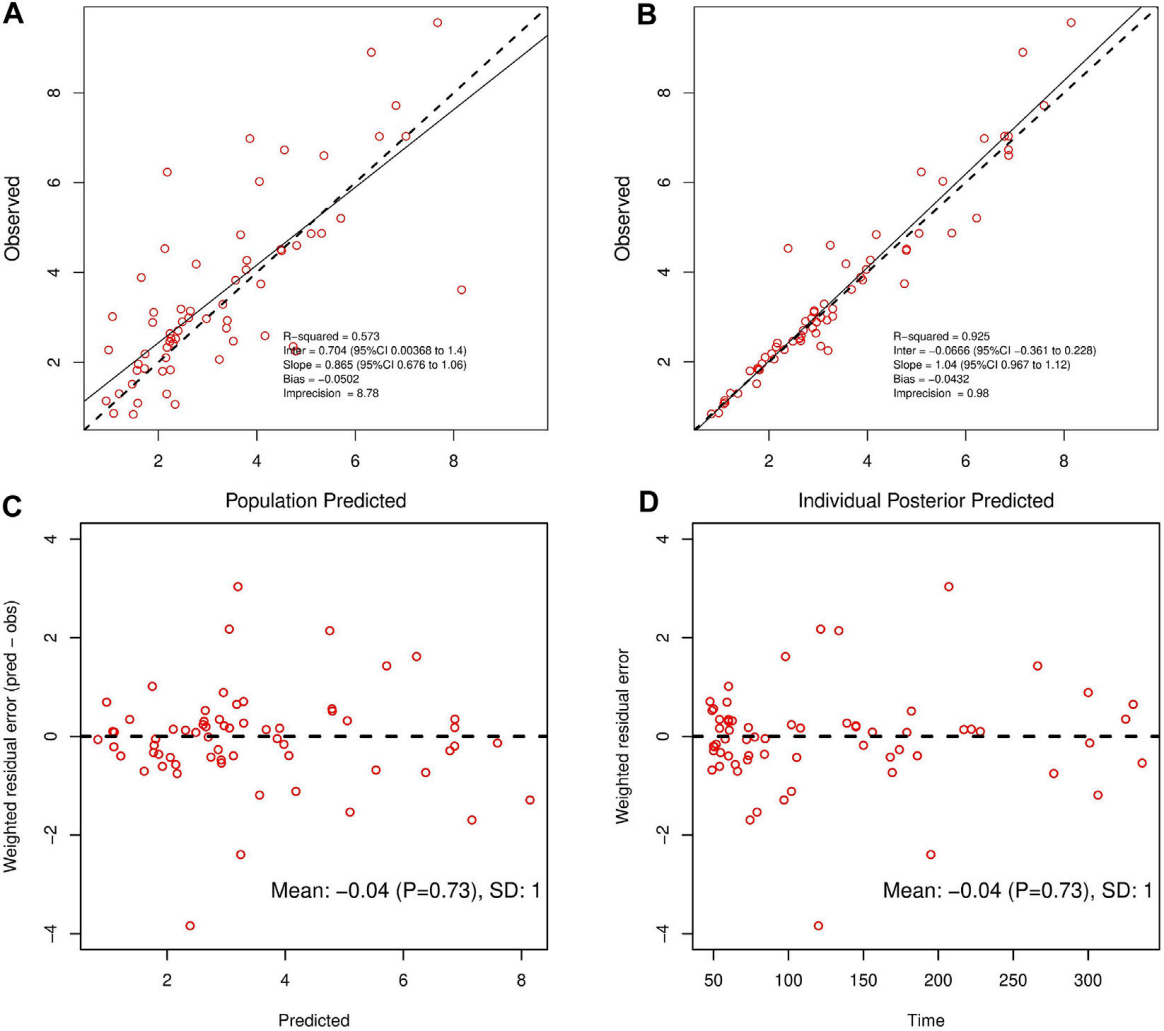
Observed concentrations versus Population Predicted **(A)** and Individual Posterior Predicted **(B)**. Weighted residuals plotted against predicted concentrations **(C)**; Weighted residuals plotted against time post-administration **(D)**.

Finally, in the model 6, after the inclusion of age and ALB in the two-compartment model, the value of—2LL decreased > 6.63 (*p* < 0.01) and the R^2^_pop_ value was enhanced to 0.573. As a result, model 6 was chosen as the final model. According to the last cycle, the gamma value was 0.72. The PK parameters of the final model for polymyxin B are shown in Table 3. The best-fit approximate value of the CL and Vc were 1.24 ± 0.38 L h^-1^ and 16.64 ± 12.74 L. Figure 2 displays the model’s goodness-of-fit graphs. According to the scatter plots for observed - population predicted concentration and observed - individual posterior predicted concentration, the model’s predicted values are concentrated along the diagonal line. At the same time, the scatter plots for weighted residual error against predicted concentration and time after administration of polymyxin B reveal that weighted residual error is primarily distributed between y = ± 2, which showed that the model has strong prediction power.

**TABLE 3:** PPK parameter estimates from model 6 for polymyxin B.

PK parameter	Final model					Bootstrap	
	Mean	Median	SD	Between-subject variability (% CV)	Shrinkage (%)	Median	95%CI
CL (L∙h^-1^)	1.24	1.22	0.38	30.48	10.83	1.22	0.94 –1.52
Q (L∙h^-1^)	3.04	2.34	2.27	74.78	13.66	2.31	1.30–4.83
Vc (L)	16.64	13.52	12.74	76.55	12.60	17.15	12.15–26.16
Vp (L)	66.2	76.53	36.25	54.76	13.60	66.34	19.89–105.32

### 3.3 Model evaluation

The data splitting method demonstrated the final model’s stability. The typical values are consistent in the complete data set, 80% subset, and 20% subset (Table 4). In the 80% subset and 20% subset, the parameters were estimated to be within ± 0.12 SD of the CL from the complete data set, ± 0.41 SD for Q, and ± 4.48 SD for Vc and ± 14.35 SD for Vp. A plot of the VPC for the final model was shown in Supplementary Figure S1.

**TABLE 4:** PPK parameter estimates and precisions of estimates (SD) for the full data set, 80% of the data, and 20% of the data.

Population parameter estimate	Cohort	Mean value	SD
CL (L∙h^-1^)	Full	1.24	0.38
	80%	1.35	0.43
	20%	1.31	0.26
Q (L∙h^-1^)	Full	3.04	2.27
	80%	3.44	2.67
	20%	2.56	1.86
V_c_ (L)	Full	16.64	12.74
	80%	17.82	13.23
	20%	13.58	8.26
V_p_ (L)	Full	66.20	36.25
	80%	40.26	41.83
	20%	64.60	21.90

### 3.4 PTA outcomes for differing ALB concentrations and ages

**FIGURE 3:**
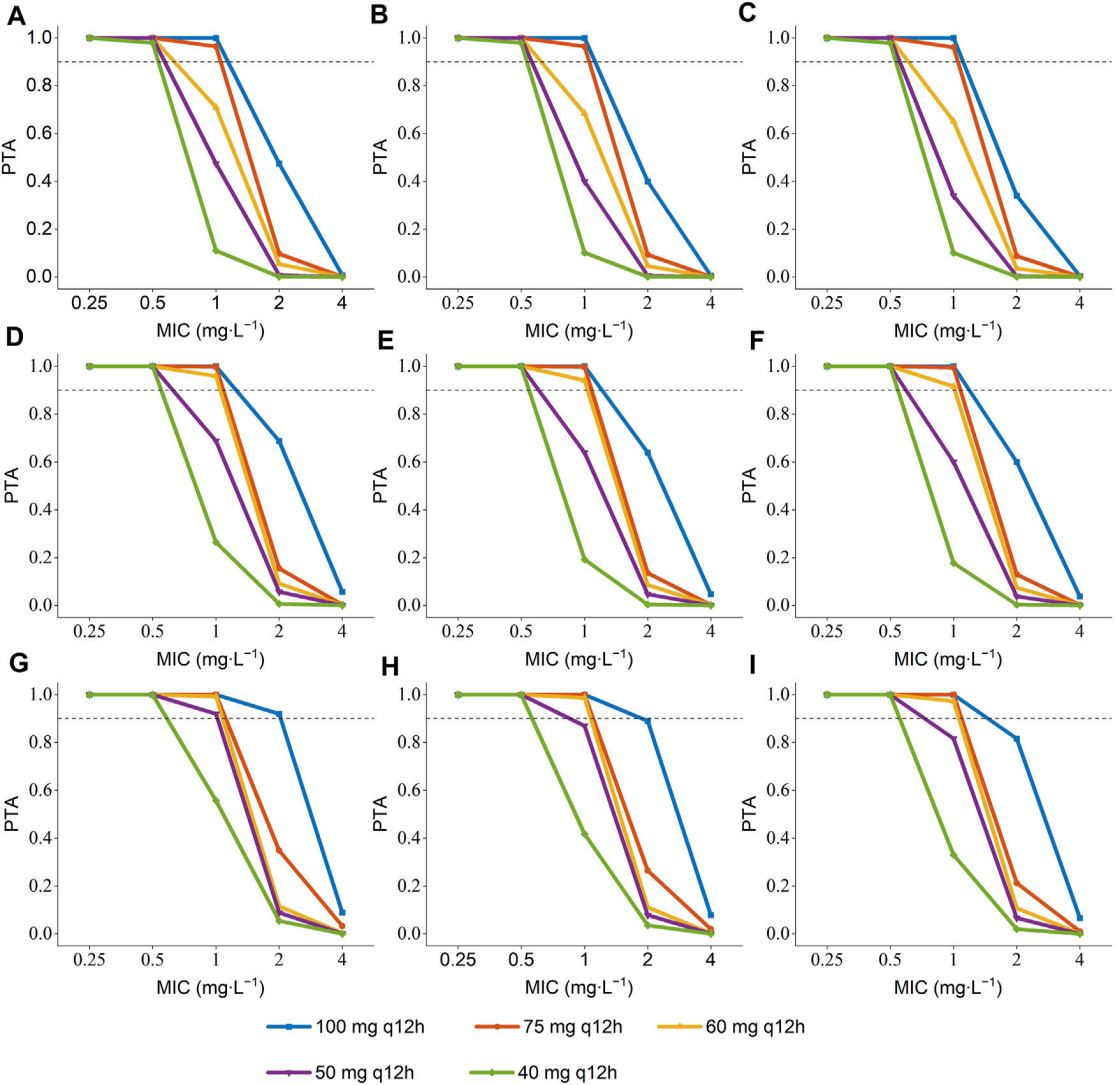
Probability of target attainment (PTA) of AUC/MIC ≥ 50 for the simulated polymyxin B dosing regimens in patients according to different ALB and age ^a^. **(A)** ALB = 23.1–24.9 g L^-1^, age = 34 years; **(B)** ALB = 23.1–24.9 g L^-1^, age = 68 years; **(C)** ALB = 23.1–24.9 g L^-1^, age = 93 years; **(D)** ALB = 25–34.9 g L^-1^, age = 34 years; **(E)** ALB = 25–34.9 g L^-1^, age = 68 years; **(F)** ALB = 25–34.9 g L^-1^, age = 93 years; **(G)** ALB = 35–41.5 g L^-1^, age = 34 years; **(H)** ALB = 35–41.5 g L^-1^, age = 68 years; **(I)** ALB = 35–41.5 g L^-1^, age = 93 years.^a^ The age were the 5th percentile, median, and 95th percentile values of the age of patients.

**FIGURE 4:**
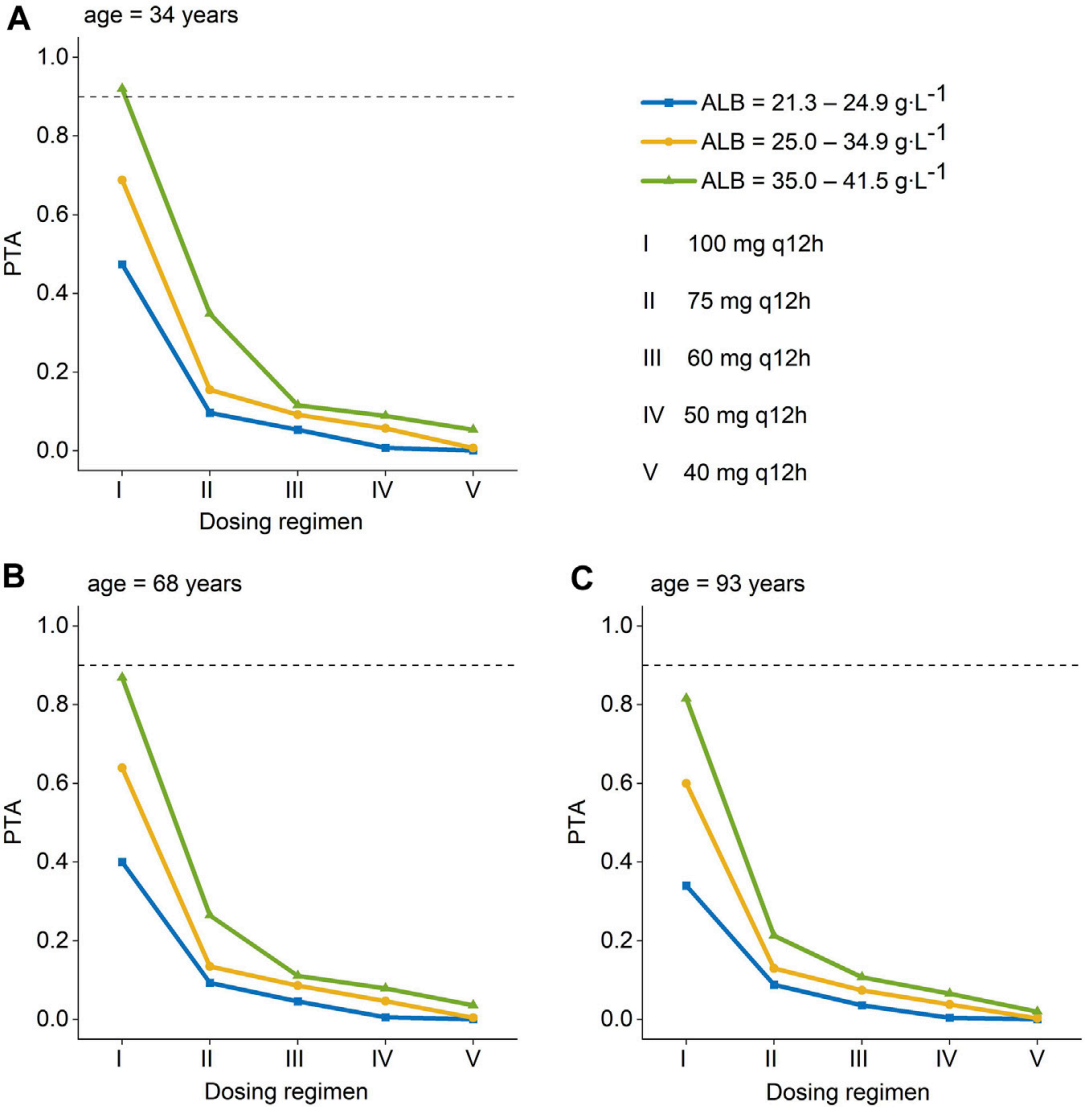
Probability of target attainment (PTA) for toxicity is defined as an AUC_ss,24h_ >100 mg∙h∙L^-1^ for polymyxin B dosing regimens administered to patients. **(A)** age = 34 years; **(B)** age = 68 years; **(C)** age = 93 years ^a^. (Ⅰ): 100 mg q12 h; (Ⅱ): 75 mg q12 h; (Ⅲ): 60 mg q12 h; (Ⅳ):50 mg q12 h; (Ⅴ):40 mg q12 h. ^a^ The age were the patients’ 5th, median, and 95th values. AUC _ss, 24h_, an area under the plasma–concentration-time curve across 24 h at steady state.

The PTA of AUC/MIC ≥ 50 at the MIC range (0.25–4 mg L^-1^) was displayed in Figure 3. When the MIC of polymyxin B against pathogenic bacteria is 1 mg L^-1^, dosing regimens should be selected according to different ALB and age. As long as the MIC is up to 1 mg L^-1^, patients in the ultra-low ALB levels group (23.1–24.9 g L^-1^) need to choose a dosing regimen of 100–75 mg q12 h (Figures 3 A, B, C); while a dosing regimen of 100–60 mg q12 h was suggested for patients in low ALB levels group (Figures 3 D, E, F). A scheme of 100–50 mg q12 h could reach efficacious PTA value in < 68 years patients with normal ALB levels, and 100–60 mg q12 h could satisfy the PTA values > 0.9 in patients aged ≥ 68 at normal ALB levels group (Figures 3 G, H, I). In addition, all dosing regimens successfully generated effective exposures at MIC ≤ 0.5 mg L^-1^. Moreover, the PTA of AUC _ss, 24h_ ≥ 100 was shown in Figure 4, and 100 mg q12 h was a toxic limit dose for patients with normal ALB levels.

## 4 Discussion

In this polymyxin B population PK study, we discovered that the ALB level was strongly associated with the CL, and the age was related to the V. Through defining these two new covariates which affect the PK parameters of polymyxin B, an outstanding contribution to optimize the clinical use of polymyxin B was made.

Since small samples were employed in this experiment, the non-parametric population modeling algorithm has theoretical advantages over parametric methods in detecting population PK/PD subgroups and outliers (Goutelle et al., 2022), so the non-parametric population modeling approach was chosen for modeling in this study. The values of CL (1.24 L h^-1^) and V (16.64 L) (Table 3) in this study were in line with the ranges from previous reports, including the CL range (1.2–4.2 L h^-1^) (Sandri et al., 2013) (Table 3) and the V range (5.63–34.4 L) (Table 5). However, the CL value was lower than that of patients accepting CVVH (4.52 L h^-1^) (Wang et al., 2022) or undergoing CRRT (1.95 L h^-1^) (Luo et al., 2022). We assumed that polymyxin B is reabsorbed back into the circulation as it passes through the renal tubules (Zavascki and Nation, 2017) while in the CRRT hemodialysis mode, there is no system in place to help polymyxin B get back into the blood from dialysis solution, which ultimately leads to increased CL. Furthermore, the CL value in this investigation was slightly higher than that of critically ill patients on ECMO (1.16 L h^-1^) (Surovoy et al., 2022), possibly due to the adsorption effect in ECMO procedures. The relevant analyses on the population PK study of polymyxin B are summarized in the following Table 5. As far as we know, a reduction in ALB levels causes a rise in the free concentration of antibiotics with a high affinity for protein, which leads to an increase in CL. Meanwhile, a thermodynamic analysis revealed a potent interaction between ALB and polymyxin B (Poursoleiman et al., 2019). It is evident from our covariate analysis that the polymyxin B’ CL lowers as the level of ALB rises. However, since the total concentration was measured rather than the free concentration, we could not determine the effect of ALB on the unbound concentration. Another covariate that was included in the population PK model was age. Previous studies have not found a significant effect of patient age on polymyxin B elimination (Chen et al., 2022). However, our study demonstrated for the first time that age appears to be a key factor impacting the V. We found the relevance between age and V (R^2^ = 0.17) and defined age as a vital element for V of polymyxin B in the covariate model.

**TABLE 5:** PPK studies of polymyxin B.

Research source	Research subjects	Number of patients(n)	Number of blood samples(n)	Pharmacokinetic model	Estimation of population parameters
Sandri et al. (2013)	Critically ill patients	24	100	two-compartment model	CL = 1.66 L h^-1^,V1 = 5.63L; Q = 8.76 L h^-1^,V2 = 19.8L
Miglis et al. (2018)	Acutely ill patients	52	156	two-compartment model	CL = 2.63 L h^-1^,V1 = 33.8L; Q = 2.32 L h^-1^,V2 = 78.2L
Kubin et al. (2018)	Clinical Samples	43	134	one-compartment model	CL = 2.37 L h^-1^,V = 34.4L
Manchandani et al. (2018)	Individuals with severe infections	35	139	one-compartment model	CL = 2.5 L h^-1^,V = 34.3L
Wang et al. (2020)	Patients with MDR-GNB infections	46	331	two-compartment model	CL = 1.786 L h^-1^,V1 = 6.218L; Q = 13.518 L h^-1^,V2 = 11.922L
Yu et al. (2021)	Patients with TDM	32	112	one-compartment model	CL = 1.59 L h^-1^,V = 20.5L
Liu et al. (2021)	Health Volunteers	20	NA	three-compartment model	CL = 1.62 L h^-1^,V = 4.26L; Q_2_ = 8.4 L h^-1^,V2 = 3.66L; Q_3_ = 0.384 L h^-1^,V3 = 2.7L
Li et al. (2021)	Renal transplant patients	50	151	one-compartment model	CL = 1.18 L h^-1^,V = 12.09L
Wang et al. (2022)	Critically ill patients with CVVH	53	275	two-compartment model	CL = 1.95 L h^-1^,V = 15.0L; Q = 2.28 L h^-1^,V2 = 6.54L; CL_CVVH_ = 4.52 L h^-1^
Luo et al. (2022)	Critically ill patients with/without CRRT	53	189	two-compartment model	CL_withoutCRRT_ = 1.5 L h^-1^,V = 11.7L; Q = 1.34 L h^-1^,V2 = 17.9L; CL_CRRT_ = 1.95 L h^-1^
Surovoy et al. (2022)	Patients with/without ECMO	13	NA	non-compartment model	CL = 1.16 L h^-1^,V = 19.73L

Since the MIC of polymyxins differs for each pathogen, for *Enterobacteriaceae, Pseudomonas aeruginosa, and Acinetobacter baumannii,* MIC = 2 mg L^-1^ is determined as the mediator value, and MIC = 4 mg L^-1^ is determined as the resistance value. The MIC value of the pathogenic strain is also an essential factor that should be considered when utilizing polymyxin B for clinical therapy. The results of Monte Carlo simulation illustrate that, for MIC of polymyxin B against bacterial strain ≤ 1 mg L^-1^, 100–75 mg q12 h should be considered for patients with ultra-low ALB stages (Figures 3 A, B, C), while 100–60 mg q12 h was recommended for patients with low ALB phases (Figures 3 D, E, F). What’s more, 100–50 mg q12 h could arrive at the target PTA of 0.9 at MIC = 1 mg L^-1^ for patients aged 34 with normal ALB levels, and 100–60 mg q12 h could fulfill the target PTA of 0.9 at MIC ≤ 1 mg L^-1^ at the age of 68 and 93 years (Figures 3 G, H, I). However, for bacteria with 2 mg L^-1^ MICs, only 100 mg q12 h meet the PTA = 0.9 for patients aged 34 with normal ALB levels (Figure 3). The PTA values for all dosing strategies were < 0.9 when the strain’s MIC value was 4 mg L^-1^. The poor bactericidal performance of dosage regimens highlights the substantial potential of antibiotic treatment failure in these circumstances (MIC ≥ 2). Moreover, to figure out the risk of polymyxin B’s toxic effects, the PTA of different dosing regimens reaching renal damage AUC_ss, 24h_ (≥100 mg∙h∙L^-1^) were shown in Figure 4. Additionally, consistent with previous results in critically ill patients (Miglis et al., 2018), 100 mg q12 h was not advised for MIC of 2 mg L^-1^ (Satlin et al., 2020), because the patients, who were 34 years old and had normal ALB levels, were at an elevated risk of nephrotoxicity (PTA of AUC_ss, 24h_ ≥ 100 mg∙h∙L^-1^ more than 0.9). In summary, we eventually advise a dosing regimen of 75 mg q12 h for most critically ill patients with MDR-GNB infections. Also, the dose of polymyxin B for critically ill older patients should not be decreased if their ALB levels are low or ultra-low.

However, there are still shortcomings in our research. On the one hand, the sample size of our research was limited, which probably made the model estimates biased. On the other hand, the final model can only be applied to the critically ill patients’ population who are not treated with CRRT or ECMO and cannot be extrapolated. Finally, we considered only 5 covariates, including age, weight, ALB, creatinine clearance, and APACHE-Ⅱ score, likely ignoring other potentially influential factors in the clinic. Therefore, the future work will expand the sample size and the influencing factors for further research.

## 5 Conclusion

This study established a polymyxin B population PK model for critically ill patients. Since age and ALB levels were discovered as two key concomitant variables, an outstanding contribution was made in directing the individualized dosing of polymyxin B in critically ill patients. The increment of ALB in patients enhanced the possibility of getting the best exposure, while the possibility of obtaining an AUC/MIC ≥ 50 decreased with older age. In order to achieve therapeutic efficacy, the dose of polymyxin B needs to be heightened for critically ill elderly patients who often have lower ALB levels.

## Data Availability

The original contributions presented in the study are included in the article/Supplementary Materials, further inquiries can be directed to the corresponding authors.
