# Supplementary material for: Population pharmacokinetic analysis and dosing optimization of polymyxin B in critically ill patients
Source: Front Pharmacol. 2023 Mar 29;14:1122310. doi: 10.3389/fphar.2023.1122310 (PMC10090446; doi:10.3389/fphar.2023.1122310)
Supplement: Supplementary file 1 [file Presentation1.pdf]

frontiers

Supplementary Material

Supplementary Figures

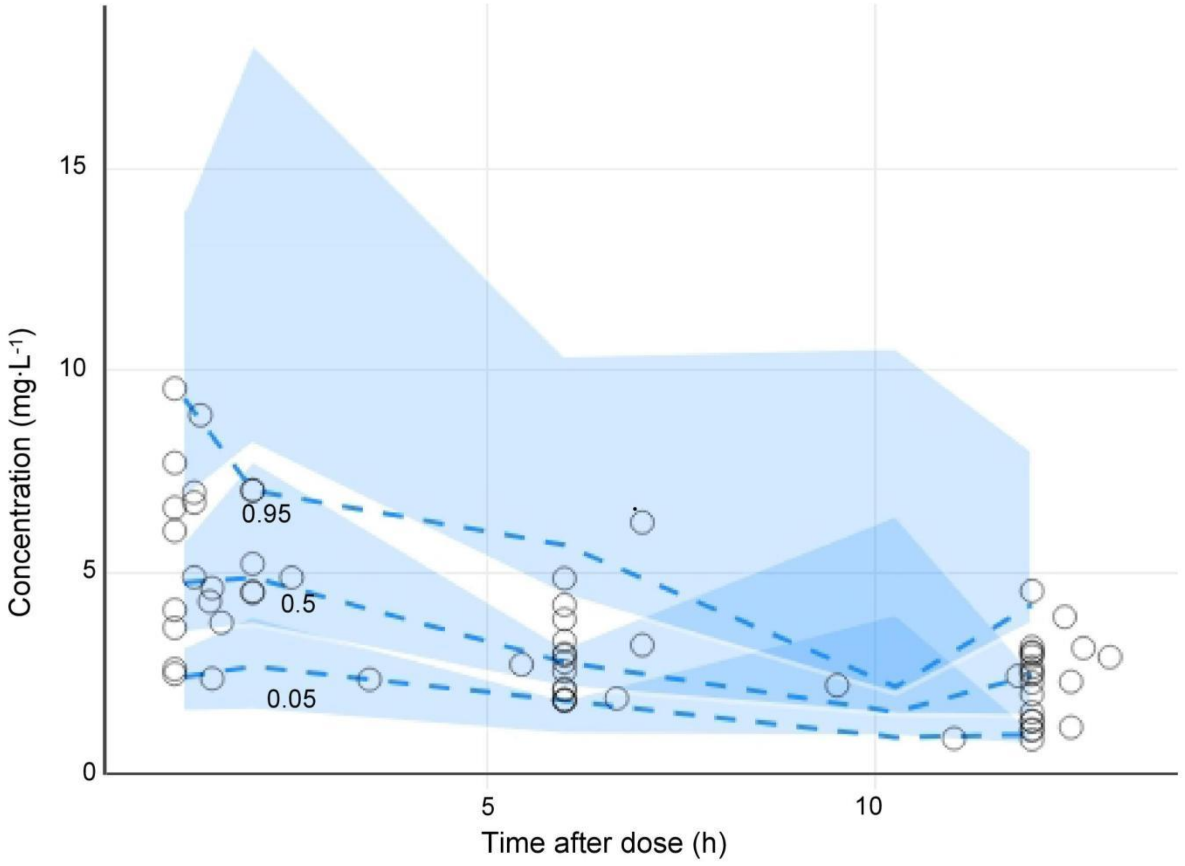

Figure S1. Visual predictive check for the final model. Open circles represent observed data.The lines representthe 5th, 50th and 95th percentiles percentiles based on simulations of the pharmacokinetic model. The shaded areas represent the 95% confidence intervals corresponding to the percentiles of the simulated data (n = 1000).
